# A Prediction Model for Identifying Seasonal Influenza Vaccination Uptake Among Children in Wuxi, China: Prospective Observational Study

**DOI:** 10.2196/56064

**Published:** 2024-06-17

**Authors:** Qiang Wang, Liuqing Yang, Shixin Xiu, Yuan Shen, Hui Jin, Leesa Lin

**Affiliations:** 1 Department of Epidemiology School of Public Health Fudan University Shanghai China; 2 Department of Epidemiology and Health Statistics School of Public Health Southeast University Nanjing China; 3 Department of Immunization Affiliated Wuxi Center for Disease Control and Prevention of Nanjing Medical University Wuxi Center for Disease Control and Prevention Wuxi China; 4 Department of Infectious Disease Epidemiology London School of Hygiene & Tropical Medicine London United Kingdom; 5 Laboratory of Data Discovery for Health (D24H) Hong Kong Science Park Hong Kong Special Administrative Region China; 6 WHO Collaborating Centre for Infectious Disease Epidemiology and Control School of Public Health, Li Ka Shing Faculty of Medicine The University of Hong Kong Hong Kong Special Administrative Region China

**Keywords:** influenza, vaccination, children, prediction model, China, vaccine, behaviors, health care professional, intervention, sociodemographics, vaccine hesitancy, clinic, Bayesian network, logistic regression, accuracy, Cohen κ, prediction, public health, immunization, digital age

## Abstract

**Background:**

Predicting vaccination behaviors accurately could provide insights for health care professionals to develop targeted interventions.

**Objective:**

The aim of this study was to develop predictive models for influenza vaccination behavior among children in China.

**Methods:**

We obtained data from a prospective observational study in Wuxi, eastern China. The predicted outcome was individual-level vaccine uptake and covariates included sociodemographics of the child and parent, parental vaccine hesitancy, perceptions of convenience to the clinic, satisfaction with clinic services, and willingness to vaccinate. Bayesian networks, logistic regression, least absolute shrinkage and selection operator (LASSO) regression, support vector machine (SVM), naive Bayes (NB), random forest (RF), and decision tree classifiers were used to construct prediction models. Various performance metrics, including area under the receiver operating characteristic curve (AUC), were used to evaluate the predictive performance of the different models. Receiver operating characteristic curves and calibration plots were used to assess model performance.

**Results:**

A total of 2383 participants were included in the study; 83.2% of these children (n=1982) were <5 years old and 6.6% (n=158) had previously received an influenza vaccine. More than half (1356/2383, 56.9%) the parents indicated a willingness to vaccinate their child against influenza. Among the 2383 children, 26.3% (n=627) received influenza vaccination during the 2020-2021 season. Within the training set, the RF model showed the best performance across all metrics. In the validation set, the logistic regression model and NB model had the highest AUC values; the SVM model had the highest precision; the NB model had the highest recall; and the logistic regression model had the highest accuracy, F1 score, and Cohen κ value. The LASSO and logistic regression models were well-calibrated.

**Conclusions:**

The developed prediction model can be used to quantify the uptake of seasonal influenza vaccination for children in China. The stepwise logistic regression model may be better suited for prediction purposes.

## Introduction

The population of China comprises over 200 million children under the age of 15 years, accounting for approximately 10% of the global population in that age band [1]. The incidence rate of influenza infections among children aged ≤14 years was estimated to be 15.86 per 1000 person-seasons, a notably elevated figure in comparison to those of younger adults (5.17) and older adults (2.37) in China [2]. In the realm of public health interventions preventing influenza transmission, vaccination stands as an important strategy. A study conducted in the United States revealed that influenza vaccination averted 2.36 million influenza-associated illnesses among children aged 6 months to 17 years in the 2018-2019 season [3]. The technical influenza vaccination guideline in China recommends the inoculation of children aged 6 to 59 months as well as school-age children against influenza [4]. Such recommendations were underpinned by the rationale that children aged 6 to 59 months are at heightened risk of developing severe symptoms subsequent to infection, whereas school-age children, owing to their extensive social interactions, might propagate the transmission of the influenza virus [5,6]. However, a meta-analysis showed that only one-quarter of children aged 6 months to 17 years in mainland China were vaccinated against influenza [7].

Identifying individuals who are unlikely to receive the influenza vaccine in the upcoming season could provide valuable insights for public health strategies. For example, this information would be essential for health care professionals and policy makers to allocate resources effectively and develop targeted interventions to increase vaccination uptake. Several efforts have been made to develop a prediction model to identify vaccination uptake behavior. Loiacono et al [8] developed and validated a logistic regression model to predict adults’ influenza vaccine uptake in England. Another logistic regression model was constructed by Oster et al [9] using birth hospitalization records to predict undervaccination status in the United States. An expanding array of methodologies has been adopted for constructing prediction models and their subsequent application within clinical contexts, including Bayesian networks (BNs) and machine learning (ML) models [10,11]. Models derived from these diverse, adaptable, and intricate techniques have demonstrated potential for enhanced predictive accuracy. However, there are limited studies examining the performance of BN and ML models in predicting vaccination behaviors.

A multitude of factors have been identified as influential in influenza vaccine uptake, encompassing sociodemographic factors (such as age and education), level of vaccine confidence, and prior vaccine experiences [12,13]. Although Loiacono et al [8] included sociodemographic factors, vaccination history, and clinical conditions from primary care records as predictors when developing their predictive model, few studies have incorporated individuals’ vaccination intentions as predictors. Individuals’ attitudes toward vaccines could potentially exert a direct impact on their vaccination behaviors [14]. Therefore, variables associated with these attitudes should be integrated into prediction models to enhance the predictive performance.

In this study, we developed predictive models of influenza vaccination behavior among children aged 6 months to 14 years using different methods (BN, ML, and regression) and assessed the performance metrics of these models.

## Methods

### Study Design and Participants

**Figure 1:**
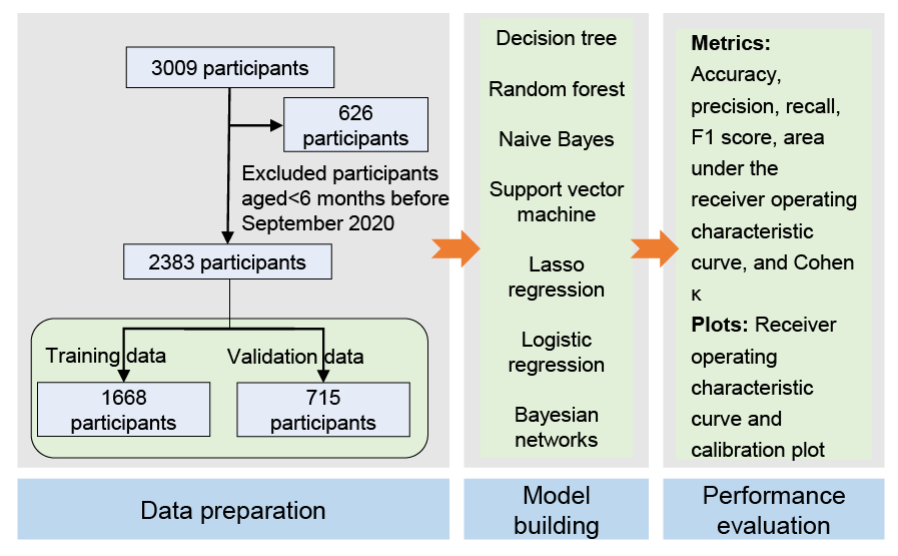
Flowchart of participant selection. Lasso: least absolute shrinkage and selection operator.

We performed a cross-section survey in Wuxi city of eastern China between September 21 and October 17, 2020. Wuxi (31.5704° N latitude and 120.3055° E longitude) has a densely populated demographic (approximately 7.46 million residents) and a well-developed transportation infrastructure, which provide conducive conditions for the spread of influenza virus. Thus, determining the factors that can accurately predict influenza vaccination behaviors has practical implications, potentially alleviating the influenza-related burden in the city. Participants were recruited from six immunization clinics, including Anzhen, Dongting, Huazhuang, Jiangxi, Meicun, and Taihu, in Wuxi between September 21 and October 17, 2020 [15]. Parents bringing their child to the immunization clinic for vaccination were encouraged to participate in our study. We excluded children lacking an immunization record number (which is used to track vaccination administration records) and children aged >14 years. A total of 3009 participants were recruited. Individuals aged ≥6 months are eligible to receive the influenza vaccine in China [4,5]. To ensure that children would be eligible for influenza vaccination (ie, aged ≥6 months) at cohort entry, we excluded children born after March 2020 (n=626) (Figure 1).

### Ethical Considerations

The Ethics Committee of Wuxi Center for Disease Control and Prevention granted approval for this study (2020No10). All participants who consented to complete the survey were required to sign informed consent forms. We assured participants that their survey responses would remain anonymous. Participants received a gift valued at 5 RMB (US $1=6.8148 RMB) upon completing the survey.

### Outcomes and Candidate Predictors

One year after the survey, we extracted the influenza vaccination records for each child, including the vaccination time and site. The immunization records were retrieved from the Jiangsu Information Management System of Vaccination Cases [16]. The predicted outcome variable was influenza vaccination status during the 2020-2021 season, specifically between October 18, 2020, and June 30, 2021 (the final date for the supply of influenza vaccines in Wuxi during the 2020-2021 season).

Data on children’s characteristics such as age, sex, and firstborn status were obtained at cohort entry. Since parents are mainly responsible for children’s vaccinations, we also collected the parents’ sociodemographic information, including age, relationship to the child, education level, annual family income, and occupation. Parental vaccine hesitancy was measured using the 10-item Vaccine Hesitancy Scale based on a 5-point Likert scale [17]. We reversed answers to items L5, L9, and L10 as they are phrased negatively. The total score for all 10 items was summed and the total maximum score was 50 points. We established a cut-off value of 40, where scores ≤40 indicated “high hesitancy” and scores >40 indicated “low hesitancy” [15]. A previous study indicated that the distance to the immunization site was associated with vaccination behavior [18]. Therefore, we investigated parents’ perceptions of convenience to the clinic and satisfaction with the clinic services using one question for each aspect rated on a 5-point Likert scale. The ratings of “strong agreement” and “agreement” were coded together into “agree” and otherwise the responses were coded as “disagree.” Parents were asked about their intention to have their children vaccinated against influenza in the 2020-2021 season, with answers of “yes,” “not sure,” or “no” possible. The responses “no” and “not sure” were both included in the category “no” in the analysis. We also developed the predictive models under the conditions with and without combining “no” and “not sure” for comparison. The prior influenza vaccination history (prior to the 2020-2021 influenza season) was extracted as a predictor from the electronic health record system. All covariates are provided in Table S1 of Multimedia Appendix 1.

### Model Building

The BN-based model was constructed using an iterative process in which the network structure was gradually constructed through a combination of manual construction and data-driven methods. Initially, a blocklist of arcs (ie, arcs that cannot be present in the network structure) was determined based on expert knowledge and logical relationships (order of event occurrences) using the variables collected. Using a scoring algorithm (the hill-climbing algorithm), 200 network structures were learned and constructed using bootstrap sampling based on all variables. The frequency of each directed arc in the 200 network structures was calculated, and arcs with frequencies higher than 60% were retained to derive an average network structure [10,19]. The variables not included in the network would then be excluded. The obtained average network structure was further optimized by removing illogical arcs (such as child’s sex to firstborn status) and adjusting the direction of arcs that did not align with the logic (such as firstborn status to parents’ education level). The resulting network structure served as the basis for Bayesian posterior estimation, enabling the computation of conditional probabilities for each node.

We considered both stepwise logistic regression and least absolute shrinkage and selection operator (LASSO) regression. In the initial construction of the logistic and LASSO regression models, all variables were included. The logistic regression model was progressively refined through backward stepwise algorithms that systematically reduced variables by minimizing the Akaike information criterion (AIC) [8]. The model with the lowest AIC would be chosen as the prediction model. For LASSO regression, variable selection was achieved by penalizing the absolute values of the coefficients of the variables. As the tuning parameter λ increases, coefficients of more variables shrink toward 0, which could result in a sparse model where only a subset of predictors are retained in the final model [8]. Through determining the optimal value of λ using 10-fold cross-validation on the training set, we could minimize the prediction error. We report the estimated coefficients of variables in the final logistic regression and LASSO regression to indicate the contribution of variables.

The support vector machine (SVM), naive Bayes (NB), random forest (RF), and decision tree classifier (DTC) algorithms were also used to construct prediction models. All variables collected were included in the SVM, NB, and RF models. Ranges were set for cost and ɣ in the SVM model and a grid search was performed within the specified ranges to optimize the hyperparameters. Initially, all variables were included in the DTC model. We calculated the cost-complexity parameter value that yielded the lowest cross-validated error and pruned the tree model accordingly (ie, some variables were excluded) to prevent overfitting in the DTC model, resulting in a new tree to predict.

### Statistical Analysis

The variables were characterized by the frequency of occurrence. The data set was randomly split into training and validation sets at a 7:3 ratio. The training set was used for constructing the prediction model, whereas the validation set was used to validate the model and assess the model’s performance. The children’s vaccination status was classified based on the predicted probability following the approach proposed by Loiacono et al [8]. Specifically, a predicted probability ≤0.50 was assigned to indicate nonreceipt of vaccination, while a probability above 0.50 indicated receipt of vaccination. Receiver operating characteristic (ROC) curves were plotted using sensitivity and 1–specificity. We evaluated model performance using various metrics, including accuracy, precision, recall, F1 score, area under the ROC curve (AUC), and Cohen κ (see Table S2 in Multimedia Appendix 1). Calibration plots were also generated to compare the predicted outcomes from the model with the observed outcomes, providing insights into the alignment between predicted and actual probabilities [20]. Additionally, we performed sensitivity analyses, excluding children who were too young to have an influenza vaccination history (aged less than 6 months in September 2019). We also developed a model for children aged 6 months to 5 years as this is highlighted as the high-risk demographic for influenza in the guidelines [4,5].

All analyses were performed using R packages (bnlearn 4.8.1 and gRain 1.3.9 for the BN and NB models, MASS 7.3-51.5 for the logistic regression, glmnet 4.1-7 for the LASSO regression, e1071 1.7-13 for the SVM model, randomForest 4.6-12 for the RF model, and rpart 4.1.19 for the DTC model). We used Netica software 6.09 [21] to perform BN inference. Statistical significance was determined at a *P* value threshold of <.05 using a two-sided test.

## Results

### Participant Characteristics

The analyses included a total of 2383 individuals. Overall, 56.9% (1356/2383) of parents expressed a willingness to vaccinate their children against influenza, whereas 26.3% (627/2383) of children had received the influenza vaccine during the 2020-2021 season. The vaccine uptake status was significantly associated with various variables, including the children’s age, firstborn status, and previous influenza vaccine history, as well as parental factors such as age, educational level, annual household income, vaccine hesitancy, and willingness to vaccinate (Table 1). Among parents expressing an unwillingness to vaccinate their child, a notable proportion of children (90/1027, 8.7%) received vaccination. Among parents indicating a willingness to vaccinate their child, a considerable proportion of children (819/1356, 60.4%) remained unvaccinated (Table S3 in Multimedia Appendix 1). These results suggest that within the subset of parents expressing a willingness to vaccinate their children, factors including younger age of children, firstborn status, and absence of prior influenza vaccination, as well as lower parental education level and household income, may pose barriers to eventual vaccine uptake. The training data set comprised 1668 children and the validation data set included 715 children. There were no significant differences in the variables between the training and validation data sets (see Table S4 in Multimedia Appendix 1).

**Table 1:** Characteristics of participants.

Characteristic			All (N=2383), n (%)	Not receiving influenza vaccine (n=1756), n (%)	Receiving influenza vaccine (n=627), n (%)	χ^2^		*df*		*P* value	
**Children**											
	**Age group**						523.02		4		<.001
		6 months-2 years	1229 (51.6)	1144 (65.1)	85 (13.6)						
		3-5 years	753 (31.6)	360 (20.5)	393 (62.7)						
		6-8 years	349 (14.6)	219 (12.5)	130 (20.7)						
		9-11 years	37 (1.6)	23 (1.3)	14 (2.2)						
		≥12 years	15 (0.6)	10 (0.6)	5 (0.8)						
	**Sex**						0.36		1		.55
		Male	1214 (50.9)	901 (51.3)	313 (49.9)						
		Female	1169 (49.1)	855 (48.7)	314 (50.1)						
	**Firstborn**						18.78		1		<.001
		Yes	1585 (66.5)	1124 (64.0)	461 (73.5)						
		No	798 (33.5)	632 (36.0)	166 (26.5)						
	**Prior influenza vaccine uptake**						226.16		1		<.001
		No	2225 (93.4)	36 (2.1)	122 (19.5)						
		Yes	158 (6.6)	1720 (97.9)	505 (80.5)						
**Parents**											
	**Relationship with child**						2.22		1		.14
		Mother	1779 (74.7)	1297 (73.9)	482 (76.9)						
		Father	604 (25.3)	459 (26.1)	145 (23.1)						
	**Age group (years)**						95.27		4		<.001
		<26	163 (6.8)	155 (8.8)	8 (1.3)						
		26-30	799 (33.5)	649 (37)	150 (23.9)						
		31-35	959 (40.2)	648 (36.9)	311 (49.6)						
		36-40	377 (15.8)	248 (14.1)	129 (20.6)						
		≥41	85 (3.6)	56 (3.2)	29 (4.6)						
	**Education level**						40.98		3		<.001
		Junior high school or below	263 (11.0)	224 (12.8)	39 (6.2)						
		High school graduate or equivalent	467 (19.6)	375 (21.4)	92 (14.7)						
		College or equivalent	1487 (62.4)	1046 (59.6)	441 (70.3)						
		Master’s degree or above	166 (7.0)	111 (6.3)	55 (8.8)						
	**Annual household income (US $)**						66.87		3		<.001
		<7669	167 (7.0)	140 (8.0)	27 (4.3)						
		7669 to <15,337	677 (28.4)	556 (31.7)	121 (19.3)						
		15,337 to <23,006	612 (25.7)	454 (25.9)	158 (25.2)						
		≥23,006	927 (38.9)	606 (34.5)	321 (51.2)						
	**Health care occupation**						0.03		1		.86
		Yes	148 (6.2)	110 (6.3)	38 (6.1)						
		No	2235 (93.8)	1646 (93.7)	589 (93.9)						
	**Vaccine hesitancy**						13.64		1		<.001
		High	154 (6.5)	133 (7.6)	21 (3.3)						
		Low	2229 (93.5)	1623 (92.4)	606 (96.7)						
	**Willingness to accept influenza vaccine**						286.65		1		<.001
		Yes	1356 (56.9)	819 (46.6)	537 (85.6)						
		No	1027 (43.1)	937 (53.4)	90 (14.4)						
	**Convenience of immunization clinic**						1.07		1		.30
		Agree	1487 (62.4)	1085 (61.8)	402 (64.1)						
		Disagree	896 (37.6)	671 (38.2)	225 (35.9)						
	**Satisfaction with immunization clinic service**						1.49		1		.22
		Agree	2326 (97.6)	1718 (97.8)	608 (97.0)						
		Disagree	57 (2.4)	38 (2.2)	19 (3.0)						

### Model Training

The final BN framework consisted of 10 nodes and 11 arcs, including children’s age, firstborn status, previous influenza vaccine history, parental age, education level, annual household income, vaccine hesitancy, willingness to vaccinate, satisfaction with clinic services, and vaccination behavior (Figure S1 in Multimedia Appendix 1). Child’s sex, relationship with child, parent’s occupation, and convenience to the clinic were not included in the BN framework. Child’s age, influenza vaccine history, and willingness to vaccinate were directly linked to the influenza vaccination behavior. Additionally, parent’s age had an indirect association with the vaccination behavior. When the BN receives information from individual findings, probabilistic reasoning is used to determine the maximum a posteriori probability of receiving the vaccine. For instance, if parents expressed a willingness to vaccinate their child and the child had been vaccinated against influenza previously, the probability of receiving the influenza vaccine increased from 26.6% to 85.6% (Figure S1 in Multimedia Appendix 1).

A total of 6 variables were ultimately included in the logistic regression model (Table S5 in Multimedia Appendix 1). The LASSO regression model (Table S5 in Multimedia Appendix 1) included 10 variables, with the tuning parameter log(λ) set at –5.438 (Figure S2 in Multimedia Appendix 1). Child’s age, prior influenza vaccine uptake, and parents’ willingness to vaccinate had the greatest contributions to the logistic and LASSO regression models according to estimated coefficient values.

### Model Performance

Model performance metrics are summarized in Table 2. Among the seven models constructed within the training set, the RF model demonstrated the best performance, with the highest values in AUC, accuracy, precision, recall, F1 score, and Cohen κ. Within the validation set, the logistic regression model and the NB model achieved the highest AUC values, the SVM model had the highest precision, and the NB model had the highest recall. The logistic regression model had the highest accuracy, F1 score, and Cohen κ values. The performance of the seven models was similar when using different methods of combining answers related to vaccination willingness (see Table S6 in Multimedia Appendix 1).

**Table 2:** Model performance metrics.

Model		AUC^a^ (95% CI)	Accuracy	Precision	Recall	*F*1 score	Cohen κ
**Training data**							
	Bayesian network	0.861 (0.842-0.881)	0.826	0.680	0.636	0.657	0.540
	Logistic regression	0.875 (0.856-0.894)	0.838	0.700	0.674	0.687	0.578
	LASSO^b^ regression	0.877 (0.858-0.895)	0.833	0.691	0.663	0.677	0.565
	Support vector machine	0.906 (0.890-0.922)	0.862	0.778	0.663	0.716	0.625
	Naive Bayes	0.870 (0.851-0.889)	0.834	0.687	0.679	0.683	0.570
	Random forest	0.942 (0.930-0.955)	0.896	0.855	0.727	0.786	0.717
	Decision tree	0.850 (0.828-0.872)	0.842	0.722	0.649	0.683	0.578
**Validation data**							
	Bayesian network	0.829 (0.793-0.865)	0.810	0.646	0.612	0.628	0.501
	Logistic regression	0.849 (0.818-0.881)	0.813	0.644	0.644	0.644	0.516
	LASSO regression	0.848 (0.816-0.880)	0.806	0.637	0.606	0.621	0.491
	Support vector machine	0.845 (0.813-0.877)	0.811	0.667	0.564	0.611	0.487
	Naive Bayes	0.849 (0.817-0.880)	0.804	0.619	0.665	0.641	0.507
	Random forest	0.825 (0.790-0.861)	0.797	0.630	0.553	0.589	0.455
	Decision tree	0.813 (0.776-0.850)	0.799	0.617	0.617	0.617	0.480

^a^AUC: area under the receiver operating characteristic curve.

^b^LASSO: least absolute shrinkage and selection operator.

Within the training set, the prediction of receiving the vaccine was well-calibrated with the observed vaccination behavior using the DTC, LASSO, and logistic regression models (Figure S3 in Multimedia Appendix 1). Within the validation data set, the LASSO and logistic regression models consistently provided more reliable predicted probabilities. For the predicted probabilities between 0.25 and 0.50, the SVM, RF, and DTC models tended to overpredict, while the NB model tended to underpredict. For predicted probabilities over 0.50, all seven models displayed a bias toward underprediction.

### Sensitivity Analysis

A total of 1699 participants aged <6 months in September 2019 were included for the sensitivity analysis (Table S7 in Multimedia Appendix 1). The RF model exhibited the best performance within the training set, achieving the highest AUC (0.944, 95% CI 0.932-0.956), accuracy (0.881), precision (0.832), recall (0.813), F1 score (0.822), and Cohen κ (0.733) values (see Figure S4 and Table S8 in Multimedia Appendix 1). Within the validation set, the LASSO regression model had the highest AUC (0.880, 95% CI 0.849-0.910), followed by the logistic regression model (0.878, 95% CI 0.848-0.909). The NB and RF models both had the highest accuracy (0.816), while the DTC model had the highest precision (0.774). The NB model achieved the highest recall, F1 score, and Cohen κ values at 0.698, 0.719, and 0.582, respectively.

Regarding the calibration of predictions, within the training set, the predictions of the DTC, LASSO regression, logistic regression, and BN models were generally well-calibrated with the observed behavior (Figure S5 in Multimedia Appendix 1). In the validation set, none of the seven models demonstrated good calibration. When the predicted probabilities were above 0.50, the DTC, BN, LASSO regression, logistic regression, and SVM models exhibited a bias toward overprediction, while the NB and RF models showed a bias toward underprediction.

A total of 1982 participants aged 6 months to 5 years were analyzed separately (Table S9 in Multimedia Appendix 1). Within the training set, the RF model also exhibited the highest values across all performance metrics (Figure S6 and Table S10 in Multimedia Appendix 1). Within the validation set, the NB model showed the highest AUC (0.884, 95% CI 0.851-0.916), accuracy (0.860), precision (0.727), and Cohen κ (0.607) values, whereas the BN model had the highest recall (0.713) and F1 score (0.701). Within the validation set, the SVM and logistic regression models showed better calibration than the other models (Figure S7 in Multimedia Appendix 1).

## Discussion

### Principal Findings

In the study, we developed models to predict the likelihood of influenza vaccination uptake among children aged 6 months to 14 years in the upcoming season. We conducted a survey in Wuxi city, eastern China, between September 21 and October 17, 2020, and extracted the participants’ influenza vaccination records after 1 year. Data from a total of 2383 participants were included in the analysis. The RF model demonstrated superior performance metrics within the training set, whereas the logistic regression model exhibited a narrow margin of performance superiority within the validation set. Both the logistic and LASSO regression models showed good calibration. Among these models, the stepwise logistic regression approach, characterized by its simplicity and favorable interpretability, emerged as a viable candidate for predictive purposes.

The factors associated with influenza vaccination included the child’s age, child’s previous influenza vaccine uptake, parental age, educational level, annual household income, parental vaccine hesitancy, and influenza vaccination willingness. Among these factors, previous vaccine uptake emerged as a strong positive factor, as supported by previous studies [12,22,23]. Individuals who personally witnessed the positive impact due to vaccination, such as a decreased likelihood of infection, were more likely to continue receiving vaccinations. To ensure the accuracy of this predictor, we obtained this variable directly from the electronic record system rather than relying on participants’ recall, thereby enhancing its reliability. Compared to children aged between 6 months and 2 years, older children were more likely to be vaccinated against influenza. This may be attributed to their enrollment in kindergarten or school, where peer influence and social networks potentially foster vaccination uptake [24]. Parents were concerned about the adverse effects of vaccination on younger children, which may also decrease the likelihood of young children receiving the influenza vaccine. Additionally, children from households with higher levels of parental education or income were more prone to receiving the influenza vaccine in our study. However, evidence regarding the relationship between parental education, income, and children’s influenza vaccination is inconsistent [22,23]. We also analyzed the factors influencing parents who expressed a willingness to vaccinate their children but ultimately did not do so. In addition, participants might be more inclined to express willingness toward influenza vaccination due to social desirability bias [16].

Our results did not reveal superior predictive performance of the ML methods over the logistic regression model, which was consistent with the outcomes of a previous systematic review by Christodoulou et al [25]. The strength of ML lies in its ability to handle high-dimensional data [26]. In instances characterized by constrained sample sizes and a limited number of variables, ML may be susceptible to overfitting. The BN model often involves capturing conditional dependencies among modeled variables. If the data set is confined or the interrelationships between variables are not accurately captured, the predictive performance of a BN model might diminish. The number of predictors and the sample size of this study were limited, which might potentially constrain the purported advantages associated with some complex methodologies such as ML and BN models. In general, ML techniques require more than 10 instances for each predictor to mitigate the risk of overfitting [26]. Nonetheless, despite satisfying this criterion, challenges such as instability and high optimism may persist [27]. The minimum sample size required for predictive models constructed with ML and BN approaches is still being explored [28,29].

In the sensitivity analysis, RF still showed the best performance within the training set and the performance metrics of seven models did not show substantial discrepancies within the test set. Additionally, we did not find any model that showed a prominent advantage in terms of calibration capability. Logistic regression might still be the most suitable tool to predict vaccination behaviors owing to its simplicity and favorable interpretability.

### Implications for Practice

Our findings offer valuable insight for practice, allowing for prediction of the likelihood of children receiving the influenza vaccine. These modeling results can enable health care professionals to tailor their strategies based on the predicted probabilities. When dealing with children who are highly likely to be vaccinated, health care workers could save time and simplify the decision-making process [8]. For example, they could leverage messaging systems or electronic letters to provide reminders [30,31] or they could inform parents about the accessibility of the influenza vaccine during medical consultations. These efforts could particularly benefit children with a low predicted probability of vaccination. Health care workers can also use various communication techniques such as motivational interviewing and improvisational theater to encourage vaccination, which are known for their persuasive and respectful features [32,33]. These approaches can help to identify barriers and increase vaccine confidence, thereby improving vaccine uptake.

### Limitations

Our study has several limitations. First, in China, adherence to mandatory vaccination requirements prior to school enrollment is imperative [34]. Noncompliance with these vaccination mandates results in exclusion from school attendance. Consequently, parents were compelled to accompany their children to immunization clinics for vaccination administration. However, the recruitment of participants solely from immunization clinics may still introduce selection bias, potentially limiting the representativeness of the study population to the general population of children. In addition, the data collection was limited to Wuxi city in eastern China. Although we performed internal validation, the model has not been externally validated in large samples or diverse locations. Second, vaccination behavior is influenced by various factors, including parental perceptions of susceptibility to influenza, the child’s health status, and vaccine policies. To enhance the performance of the model, it is important to explore additional factors that contribute to vaccination behavior and incorporate them into the modeling. Third, influenza vaccination willingness may vary across seasons and our study focused on a specific time frame. To account for this variability, future research should consider conducting season-specific surveys to capture the changing dynamics of vaccination willingness.

### Conclusion

Our findings indicate that the influenza vaccination behavior for children aged 6 months to 14 years could be identified using the established predictive model. We propose that the stepwise logistic regression model with high accuracy and a straightforward modeling methodology could be better suited for such prediction tasks. Further validation of the model in larger and more diverse samples is necessary. More critical predictors should be considered to increase the accuracy and reliability of predicting influenza vaccination behavior among children.

## Appendix

Multimedia Appendix 1 Table S1. Candidate variables for construction of the prediction model; Table S2. Explanation of metrics evaluating model performance; Table S3. Factors influencing the gap between willingness and behaviors; Table S4. Characteristics of participants in the training and validation data sets; Figure S1. Final BN for the prediction of receiving the influenza vaccine; Table S5. Estimated coefficients in the logistic and LASSO regressions; Table S6. Model performance; Figure S2. Binomial deviance versus log(λ) for 10-fold within-sample cross-validation of the LASSO model; Figure S3: Calibration plots for training and validation data sets; Table S7. Characteristics of participants (children aged less than 6 months on September, 2019); Figure S4. Receiver operating characteristic curves of various prediction models (children aged less than 6 months on September, 2019); Table S8. Model performance (children aged less than 6 months on September, 2019); Figure S5. Calibration plots for training and validation data sets (children aged less than 6 months on September, 2019); Table S9. Characteristics of participants (children aged 6 months to 5 years); Figure S6. Receiver operating characteristic curves of various prediction models (children aged 6 months to 5 years); Table S10. Model performance (children aged 6 months to 5 years); Figure S7. Calibration plots for training and validation data sets (children aged 6 months to 5 years).

## Abbreviations

AIC: Akaike information criterion
AUC: area under the receiver operating characteristic curve
BN: Bayesian network
DTC: decision tree classifier
LASSO: least absolute shrinkage and selection operator
ML: machine learning
NB: naive Bayes
RF: random forest
ROC: receiver operating characteristic
SVM: support vector machine

## Data Availability

Data may be available from the corresponding author on reasonable request depending on the policy and procedures of the institutions that participate in the consortiums.
