# Supplementary material for: A Prediction Model for Identifying Seasonal Influenza Vaccination Uptake Among Children in Wuxi, China: Prospective Observational Study
Source: JMIR Public Health Surveill. 2024 Jun 17;10:e56064. doi: 10.2196/56064 (PMC11217706; doi:10.2196/56064)
Supplement: Multimedia Appendix 1 [file publichealth_v10i1e56064_app1.docx]

**Multimedia Appendix 1**

**Table S1. Candidate variables for the construction of prediction model**

| **Variables** | **Cutoff value(s)** |
| --- | --- |
| Children |  |
| Age group | 6 months-2 years; 3-5 years; 6-8 years; 9-11 years; ≥12 years |
| Sex | Male; Female |
| Firstborn | Yes; No |
| Prior influenza vaccine uptake | Yes; No |
| Receiving vaccine in 2020-2021 season | Yes; No |
| Parents |  |
| Relationship with child | Mother; Father |
| Age group | <26 years; 26-30 years; 31-35 years; 36-40 years; ≥41 years |
| Educational level | Junior high school or below; High school graduate or equivalent; College or equivalent; Master's Diploma or above |
| Annual household income | <50,000 RMB; 50,000-99,999 RMB; 100,000-149,999 RMB; ≥150,000 RMB |
| Healthcare occupation | Yes; No |
| Vaccine hesitancy | High; Low |
| Willingness to influenza vaccine | Yes; No |
| Convenience to immunization clinic | Agreement; Disagreement |
| Satisfaction to immunization clinic service | Agreement; Disagreement |

**Table S2. Explanation to metrics evaluating model's performance**

| **Metrics** | **Explanation** | **Reference** |
| --- | --- | --- |
| Accuracy | Accuracy measures the proportion of correctly classified instances out of the total number of instances. It is calculated as the number of true positive and true negative predictions divided by the total number of predictions. | [1,2] |
| Precision | Precision measures the proportion of true positive predictions out of all positive predictions made by the model. It is calculated as the number of true positive predictions divided by the sum of true positive and false positive predictions. | [2] |
| Recall | Recall measures the proportion of true positive predictions out of all actual positive instances in the dataset. It is calculated as the number of true positive predictions divided by the sum of true positive and false negative predictions. | [2,3] |
| F1 score | The F1 score is the harmonic mean of precision and recall. It provides a single score that balances both precision and recall. It is calculated as 2 * (precision * recall) / (precision + recall). | [2] |
| AUC | AUC measures the area under the receiver operating characteristic curve, which plots the true positive rate (recall) against the false positive rate at various thresholds. AUC provides an aggregate measure of a model's performance across all possible classification thresholds. | [2,4] |
| Cohen's Kappa | Cohen's Kappa measures the agreement between two raters (or in our study, between actual and predicted observations) while accounting for the agreement that could occur by chance. It is calculated as (observed agreement - expected agreement) / (1 - expected agreement), where observed agreement is calculated as (number of true positive predictions + number of true negative predictions)/number of all observations, and expected agreement is calculated as [(number of actual positive observations * number of predicted positive predictions / (number of all observations * number of all observations)] + [(number of actual negative observations * number of predicted negative predictions / (number of all observations * number of all observations)]. | [2] |

[1] Vihinen M. How to evaluate performance of prediction methods? Measures and their interpretation in variation effect analysis. BMC Genomics. 2012;13 Suppl 4(Suppl 4):S2.

[2] Rainio O, Teuho J, Klén R. Evaluation metrics and statistical tests for machine learning. Sci Rep. 2024;14(1):6086.

[3] Dehmer M, Basak SC. Statistical and Machine Learning Approaches for Network Analysis. Wiley, 2012.

[4] Steyerberg EW, Vickers AJ, Cook NR, et al. Assessing the performance of prediction models: a framework for traditional and novel measures. Epidemiology. 2010;21(1):128-138.

**Table S3. Factors influencing the gap between willingness and behaviors***

| **Characteristic** | **Refusal to receive the influenza vaccine** | | **Willingness to receive the influenza vaccine** | | **χ^2^** | ***P* value** |
| --- | --- | --- | --- | --- | --- | --- |
|  | **No receiving vaccine**  **(n=937)** | **Receiving the vaccine**  **(n=90)** | **No receiving vaccine**  **(n=819)** | **Receiving the vaccine**  **(n=537)** |  |  |
| **Children** |  |  |  |  |  |  |
| **Age group** |  |  |  |  | 536.27 | <0.001 |
| 6m-2 years | 618 (66.0%) | 16 (17.8%) | 526 (64.2%) | 69 (12.8%) |  |  |
| 3-5 years | 193 (20.6%) | 56 (62.2%) | 167 (20.4%) | 337 (62.8%) |  |  |
| 6-8 years | 117 (12.5%) | 18 (20.0%) | 102 (12.5%) | 112 (20.9%) |  |  |
| 9-11 years | 7 (0.7%) | 0 (0.0%) | 16 (2.0%) | 14 (2.6%) |  |  |
| ≥12 years | 2 (0.2%) | 0 (0.0%) | 8 (1.0%) | 5 (0.9%) |  |  |
| **χ^2^** | 93.94 | | 366.86 | |  |  |
| ***P* value** | <0.001 | | <0.001 | |  |  |
| **Sex** |  |  |  |  | 3.20 | 0.361 |
| Male | 486 (51.9%) | 52 (57.8%) | 415 (50.7%) | 261 (48.6%) |  |  |
| Female | 451 (48.1%) | 38 (42.2%) | 404 (49.3%) | 276 (51.4%) |  |  |
| **χ^2^** | 1.15 | | 0.56 | |  |  |
| ***P* value** | 0.284 | | 0.456 | |  |  |
| **Firstborn** |  |  |  |  | 20.54 | <0.001 |
| Yes | 587 (62.6%) | 65 (72.2%) | 537 (65.6%) | 396 (73.7%) |  |  |
| No | 350 (37.4%) | 25 (27.8%) | 282 (34.4%) | 141 (26.3%) |  |  |
| **χ^2^** | 3.25 | | 10.10 | |  |  |
| ***P* value** | 0.072 | | 0.001 | |  |  |
| **Prior influenza vaccine uptake** |  |  |  |  | 22.64 | <0.001 |
| No | 917 (97.9%) | 71 (78.9%) | 803 (98.0%) | 434 (80.8%) |  |  |
| Yes | 20 (2.1%) | 19 (21.1%) | 16 (2.0%) | 103 (19.2%) |  |  |
| **χ^2^** | 80.94 | | 120.23 | |  |  |
| ***P* value** | <0.001 | | <0.001 | |  |  |
| **Parents** |  |  |  |  |  |  |
| **Relationship with child** |  |  |  |  | 11.73 | 0.008 |
| Mother | 678 (72.4%) | 59 (65.6%) | 619 (75.6%) | 423 (78.8%) |  |  |
| Father | 259 (27.6%) | 31 (34.4%) | 200 (24.4%) | 114 (21.2%) |  |  |
| **χ^2^** | 1.88 | | 1.86 | |  |  |
| ***P* value** | 0.171 | | 0.173 | |  |  |
| **Age group** |  |  |  |  | 100.22 | <0.001 |
| <26 years | 87 (9.3%) | 2 (2.2%) | 68 (8.3%) | 6 (1.1%) |  |  |
| 26-30 years | 356 (38.0%) | 23 (25.6%) | 293 (35.8%) | 127 (23.6%) |  |  |
| 31-35 years | 344 (36.7%) | 47 (52.2%) | 304 (37.1%) | 264 (49.2%) |  |  |
| 36-40 years | 122 (13.0%) | 15 (16.7%) | 126 (15.4%) | 114 (21.2%) |  |  |
| ≥41 years | 28 (3.0%) | 3 (3.3%) | 28 (3.4%) | 26 (4.8%) |  |  |
| **χ^2^** | 14.21 | | 65.22 | |  |  |
| ***P* value** | 0.007 | | <0.001 | |  |  |
| **Educational level** |  |  |  |  | 50.40 | <0.001 |
| Junior high school or below | 133 (14.2%) | 9 (10.0%) | 91 (11.1%) | 30 (5.6%) |  |  |
| High school graduate or equivalent | 199 (21.2%) | 16 (17.8%) | 176 (21.5%) | 76 (14.2%) |  |  |
| College or equivalent | 550 (58.7%) | 61 (67.8%) | 496 (60.6%) | 380 (70.8%) |  |  |
| Master's Diploma or above | 55 (5.9%) | 4 (4.4%) | 56 (6.8%) | 51 (9.5%) |  |  |
| **χ^2^** | 2.94 | | 28.62 | |  |  |
| ***P* value** | 0.401 | | <0.001 | |  |  |
| **Annual household income** |  |  |  |  | 86.43 | <0.001 |
| <50,000 RMB | 74 (7.9%) | 5 (5.6%) | 66 (8.1%) | 22 (4.1%) |  |  |
| 50,000-99,999 RMB | 305 (32.6%) | 28 (31.1%) | 251 (30.6%) | 93 (17.3%) |  |  |
| 100,000-149,999 RMB | 266 (28.4%) | 18 (20.0%) | 188 (23.0%) | 140 (26.1%) |  |  |
| ≥150,000 RMB | 292 (31.2%) | 39 (43.3%) | 314 (38.3%) | 282 (52.5%) |  |  |
| **χ^2^** | 6.50 | | 46.69 | |  |  |
| ***P* value** | 0.090 | | <0.001 | |  |  |
| **Healthcare occupation** |  |  |  |  | 0.994 | 0.803 |
| Yes | 62 (6.6%) | 7 (7.8%) | 48 (5.9%) | 31 (5.8%) |  |  |
| No | 875 (93.4%) | 83 (92.2%) | 771 (94.1%) | 506 (94.2%) |  |  |
| **χ^2^** | 0.18 | | 0.01 | |  |  |
| ***P* value** | 0.674 | | 0.946 | |  |  |
| **Vaccine hesitancy** |  |  |  |  | 42.17 | <0.001 |
| High | 839 (89.5%) | 89 (98.9%) | 784 (95.7%) | 517 (96.3%) |  |  |
| Low | 98 (10.5%) | 1 (1.1%) | 35 (4.3%) | 20 (3.7%) |  |  |
| **χ^2^** | 8.24 | | 0.25 | |  |  |
| ***P* value** | 0.004 | | 0.616 | |  |  |
| **Convenience to immunization clinic** |  |  |  |  | 1.82 | 0.610 |
| Agreement | 579 (61.8%) | 54 (60.0%) | 506 (61.8%) | 348 (64.8%) |  |  |
| Disagreement | 358 (38.2%) | 36 (40.0%) | 313 (38.2%) | 189 (35.2%) |  |  |
| **χ^2^** | 0.11 | | 1.27 | |  |  |
| ***P* value** | 0.738 | | 0.260 | |  |  |
| **Satisfaction to immunization clinic service** |  |  |  |  | 2.55 | 0.467 |
| Agreement | 918 (98.0%) | 86 (95.6%) | 800 (97.7%) | 522 (97.2%) |  |  |
| Disagreement | 19 (2.0%) | 4 (4.4%) | 19 (2.3%) | 15 (2.8%) |  |  |
| **χ^2^** | 2.19 | | 0.30 | |  |  |
| ***P* value** | 0.139 | | 0.586 | |  |  |

*Two-by-two comparisons were performed using the Bonferroni method to correct for the statistical significance level, which was corrected to 0.05/6 = 0.0083.

**Table S4. Characteristics of participants in training and validation dataset**

| **Characteristic** | **Training data (n=1,668)** | **Validation data (n=715)** | **χ^2^** | **P value** |
| --- | --- | --- | --- | --- |
| **Children** |  |  |  |  |
| **Age group** |  |  |  |  |
| 6 months-2 years | 867 (52.0%) | 362 (50.6%) | 9.327 | 0.053 |
| 3-5 years | 536 (32.1%) | 217 (30.3%) |  |  |
| 6-8 years | 238 (14.3%) | 111 (15.5%) |  |  |
| 9-11 years | 19 (1.1%) | 18 (2.5%) |  |  |
| ≥12 years | 8 (0.5%) | 7 (1.0%) |  |  |
| **Sex** |  |  |  |  |
| Male | 871 (52.5%) | 343 (48.0%) | 3.611 | 0.057 |
| Female | 797 (47.8%) | 372 (52.0%) |  |  |
| **Firstborn** |  |  |  |  |
| Yes | 1,107 (66.4%) | 478 (66.9%) | 0.053 | 0.818 |
| No | 561 (33.6%) | 237 (33.1%) |  |  |
| **Prior influenza vaccine uptake** |  |  |  |  |
| Yes | 107 (6.4%) | 51 (7.1%) | 0.417 | 0.519 |
| No | 1,561 (93.6%) | 664 (92.9%) |  |  |
| **Influenza vaccine uptake in 20-21 season** |  |  |  |  |
| Yes | 439 (26.3%) | 188 (26.3%) | 0.000 | 0.990 |
| No | 1,229 (73.7%) | 527 (73.7%) |  |  |
| **Parents** |  |  |  |  |
| **Relationship with child** |  |  |  |  |
| Mother | 1,242 (74.5%) | 537 (75.1%) | 0.110 | 0.740 |
| Father | 426 (25.5%) | 178 (24.9%) |  |  |
| **Age group** |  |  |  |  |
| < 26 years | 120 (7.2%) | 43 (6.0%) | 5.071 | 0.280 |
| 26-30 years | 569 (34.1%) | 230 (32.2%) |  |  |
| 31-35 years | 663 (39.7%) | 296 (41.4%) |  |  |
| 36-40 years | 264 (15.8%) | 113 (15.8%) |  |  |
| ≥41 years | 52 (3.1%) | 33 (4.6%) |  |  |
| **Educational level** |  |  |  |  |
| Junior high school or below | 179 (10.7%) | 84 (11.7%) | 1.976 | 0.577 |
| High school graduate or equivalent | 319 (19.1%) | 148 (20.7%) |  |  |
| College or equivalent | 1,056 (63.3%) | 431 (60.3%) |  |  |
| Master's Diploma or above | 114 (6.8%) | 52 (7.3%) |  |  |
| **Annual household income** |  |  |  |  |
| <50,000 RMB | 104 (6.2%) | 63 (8.8%) | 5.731 | 0.126 |
| 50,000-99,999 RMB | 485 (29.1%) | 192 (26.9%) |  |  |
| 100,000-149,999 RMB | 432 (25.9%) | 180 (25.2%) |  |  |
| ≥150,000 RMB | 647 (38.8%) | 280 (39.2%) |  |  |
| **Healthcare occupation** |  |  |  |  |
| Yes | 112 (6.7%) | 36 (5.0%) | 2.424 | 0.119 |
| No | 1,556 (93.3%) | 679 (95.0%) |  |  |
| **Vaccine hesitancy** |  |  |  |  |
| High | 103 (6.2%) | 51 (7.1%) | 0.760 | 0.383 |
| Low | 1,565 (93.8%) | 664 (92.9%) |  |  |
| **Willingness to influenza vaccine** |  |  |  |  |
| Yes | 942 (56.5%) | 414 (57.9%) | 0.416 | 0.519 |
| No | 726 (43.5%) | 301 (42.1%) |  |  |
| **Convenience to immunization clinic** |  |  |  |  |
| Agreement | 1,041 (62.4%) | 446 (62.4%) | 0.000 | 0.988 |
| Disagreement | 627 (37.6%) | 269 (37.6%) |  |  |
| **Satisfaction to immunization clinic service** |  |  |  |  |
| Agreement | 1,626 (97.5%) | 700 (97.9%) | 0.378 | 0.539 |
| Disagreement | 42 (2.5%) | 15 (2.1%) |  |  |


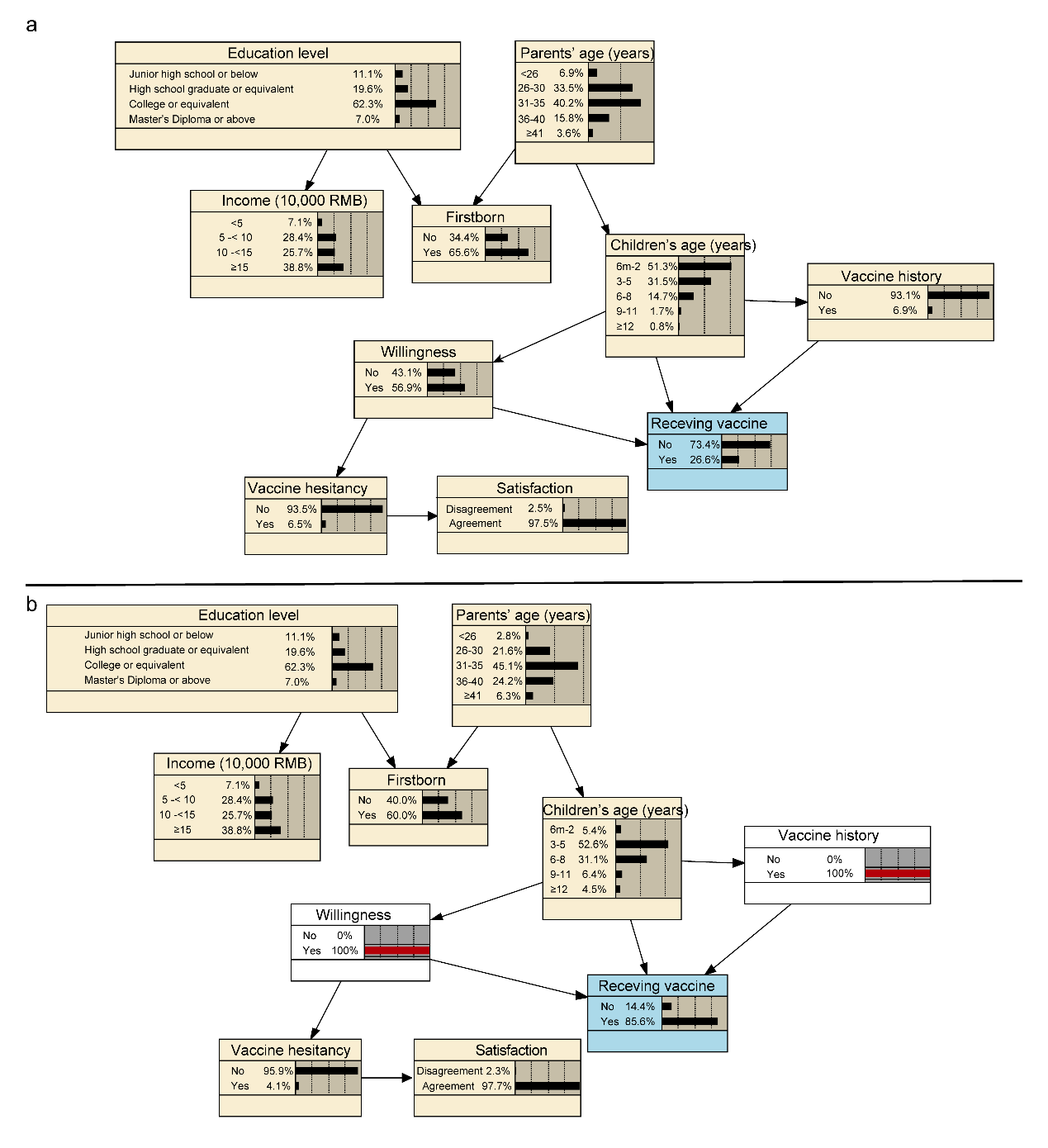


**Figure S1. Final BN for the prediction of receiving influenza vaccine**

**Table S5. Estimated coefficients in the logistic and Lasso regression**

| **Characteristic** | **Logistic** | **Lasso** |
| --- | --- | --- |
| **Children** |  |  |
| **Age group (6 months-2 years as reference)** |  |  |
| 3-5 years | 2.628 | 2.458 |
| 6-8 years | 1.967 | 1.743 |
| 9-11 years | 1.460 | 1.192 |
| ≥12 years | 0.344 | NA |
| **Sex (Male as reference)** |  |  |
| Female | NA | NA |
| **Firstborn (No as reference)** |  |  |
| Yes | NA | 0.100 |
| **Prior influenza vaccine uptake (No as reference)** |  |  |
| Yes | 2.137 | 2.034 |
| **Parents** |  |  |
| **Relationship with child (Mother as reference)** |  |  |
| Father | NA | 0.108 |
| **Age group (<26 years as reference)** |  |  |
| 26-30 years | NA | NA |
| 31-35 years | NA | 0.207 |
| 36-40 years | NA | NA |
| ≥41 years | NA | -0.130 |
| **Educational level (Junior high school or below as reference)** |  |  |
| High school graduate or equivalent | 0.165 | NA |
| College or equivalent | 0.611 | 0.327 |
| Master's Diploma or above | 0.615 | 0.231 |
| **Annual household income (<50,000 RMB as reference)** |  |  |
| 50,000-99,999 RMB | -0.145 | -0.137 |
| 100,000-149,999 RMB | 0.065 | NA |
| ≥150,000 RMB | 0.324 | 0.259 |
| **Healthcare occupation (No as reference)** |  |  |
| Yes | NA | NA |
| **Vaccine hesitancy (Low as reference)** |  |  |
| High | -0.863 | -0.675 |
| **Willingness to influenza vaccine (No as reference)** |  |  |
| Yes | 1.810 | 1.720 |
| **Convenience to immunization clinic (Disagreement as reference)** |  |  |
| Agreement | NA | 0.085 |
| **Satisfaction to immunization clinic service (Disagreement as reference)** |  |  |
| Agreement | NA | NA |

**Table S6. Model performance***

| **Model** | **AUC and 95% CI** | **Accuracy** | **Precision** | **Recall** | **F1 score** | **Cohen’s kappa** |
| --- | --- | --- | --- | --- | --- | --- |
| **Training data** |  |  |  |  |  |  |
| Bayesian network | 0.846 (0.826, 0.867) | 0.816 | 0.637 | 0.699 | 0.667 | 0.540 |
| Logistic regression | 0.867 (0.848, 0.887) | 0.826 | 0.692 | 0.613 | 0.650 | 0.535 |
| Lasso regression | 0.866 (0.847, 0.886) | 0.821 | 0.669 | 0.631 | 0.649 | 0.529 |
| Support vector machine | 0.900 (0.884, 0.917) | 0.847 | 0.749 | 0.626 | 0.682 | 0.582 |
| Naive Bayes | 0.862 (0.841, 0.882) | 0.820 | 0.655 | 0.667 | 0.661 | 0.539 |
| Random forest | 0.937 (0.923, 0.952) | 0.892 | 0.840 | 0.729 | 0.780 | 0.709 |
| Decision tree | 0.842 (0.820, 0.865) | 0.845 | 0.723 | 0.667 | 0.694 | 0.591 |
| **Validation data** |  |  |  |  |  |  |
| Bayesian network | 0.846 (0.815, 0.877) | 0.806 | 0.612 | 0.713 | 0.658 | 0.524 |
| Logistic regression | 0.866 (0.838, 0.895) | 0.817 | 0.661 | 0.622 | 0.641 | 0.518 |
| Lasso regression | 0.869 (0.840, 0.897) | 0.825 | 0.667 | 0.670 | 0.668 | 0.550 |
| Support vector machine | 0.862 (0.833, 0.891) | 0.822 | 0.694 | 0.580 | 0.632 | 0.516 |
| Naive Bayes | 0.878 (0.851, 0.906) | 0.839 | 0.680 | 0.734 | 0.706 | 0.595 |
| Random forest | 0.850 (0.817, 0.883) | 0.821 | 0.681 | 0.601 | 0.638 | 0.520 |
| Decision tree | 0.841 (0.807, 0.875) | 0.822 | 0.665 | 0.654 | 0.660 | 0.539 |

*The analyses were performed in the condition of that we did not combine “no” and “not sure” in the responses to vaccination intentions.


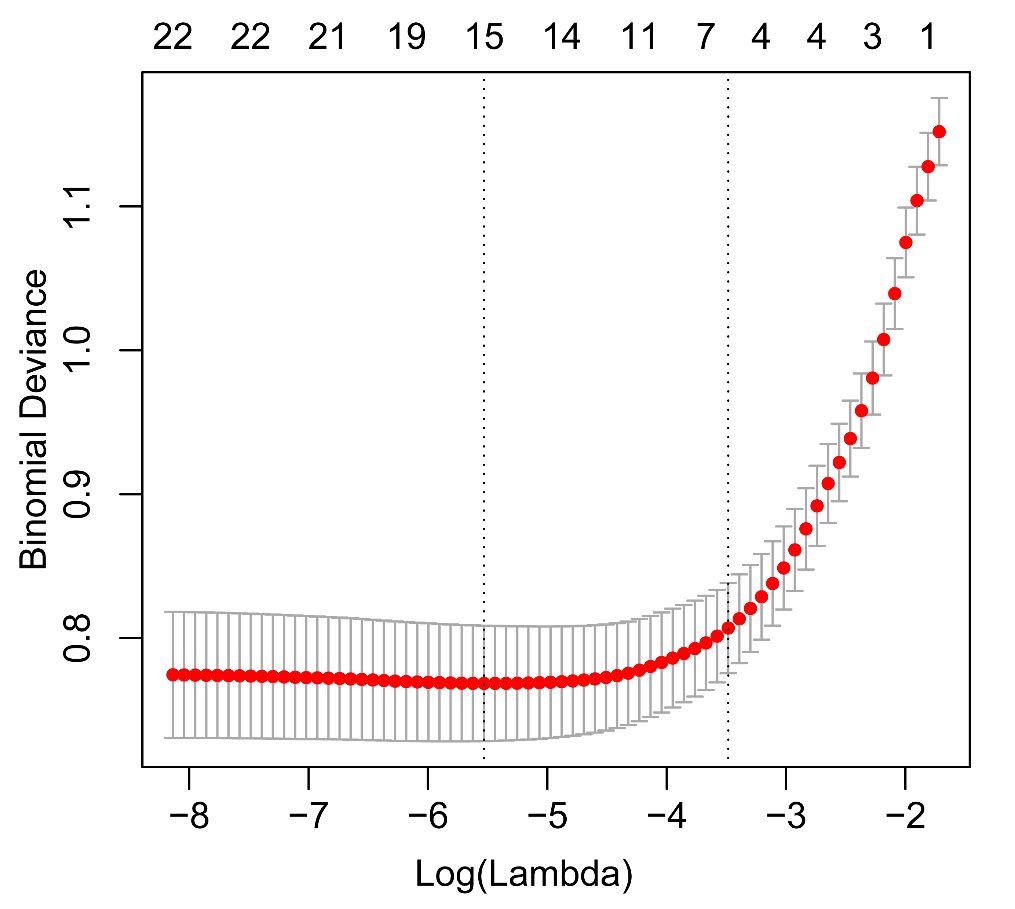


**Figure S2. Binomial deviance versus log(Lambda) for 10-fold within-sample cross-validation of LASSO model**


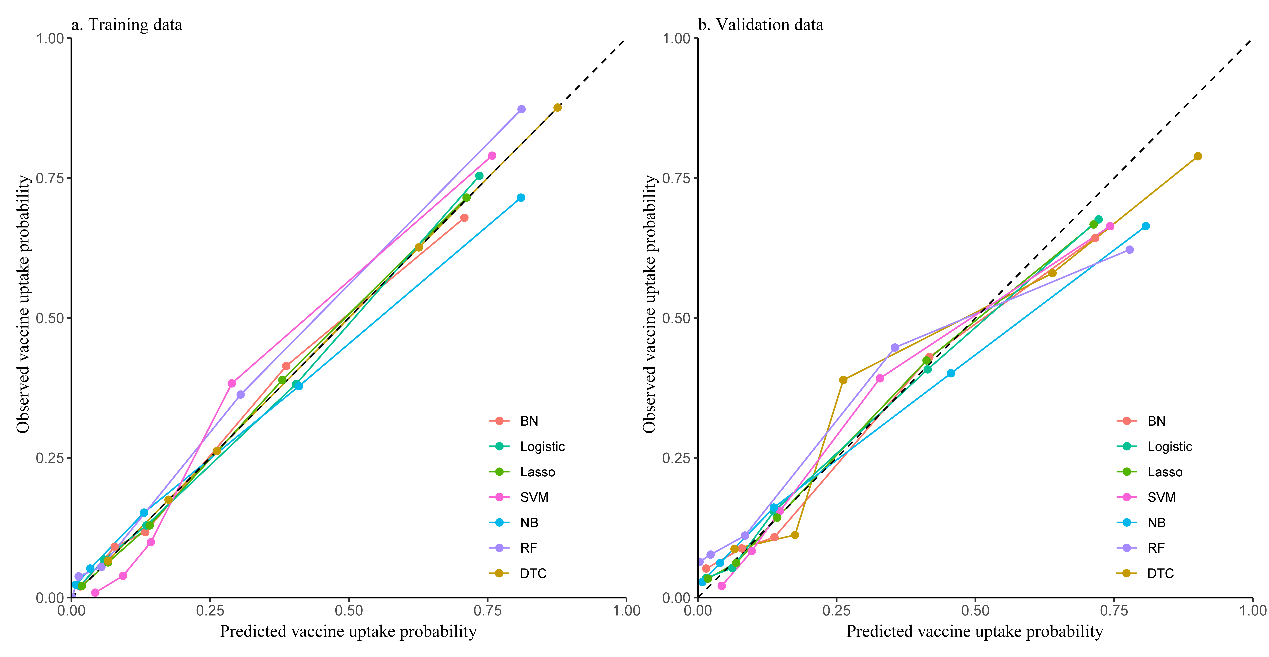


**Figure S3.** **Calibration Plots for training and validation dataset**

**Table S7. Characteristics of participants (children aged less than six months on September, 2019)**

| **Characteristic** | **All data**  **(n=1,699)** | **Training data (n=1,189)** | **Validation data (n=510)** | **χ^2^** | **P value** |
| --- | --- | --- | --- | --- | --- |
| **Children** |  |  |  |  |  |
| **Age group** |  |  |  |  |  |
| 6 months-2 years | 545 (32.1%) | 387 (32.5%) | 158 (31.0%) | 4.241 | 0.374 |
| 3-5 years | 753 (44.3%) | 537 (45.2%) | 216 (42.4%) |  |  |
| 6-8 years | 349 (20.5%) | 230 (19.3%) | 119 (23.3%) |  |  |
| 9-11 years | 37 (2.2%) | 24 (2.0%) | 13 (2.5%) |  |  |
| ≥12 years | 15 (0.9%) | 11 (0.9%) | 4 (0.8%) |  |  |
| **Sex** |  |  |  |  |  |
| Male | 866 (51.0%) | 597 (50.2%) | 269 (52.7%) | 0.918 | 0.338 |
| Female | 833 (49.0%) | 592 (49.8%) | 241 (47.3%) |  |  |
| **Firstborn** |  |  |  |  |  |
| Yes | 1,137 (66.9%) | 799 (67.2%) | 338 (66.3%) | 0.138 | 0.710 |
| No | 562 (33.1%) | 390 (32.8%) | 172 (33.7%) |  |  |
| **Prior influenza vaccine uptake** |  |  |  |  |  |
| Yes | 157 (9.2%) | 104 (8.7%) | 53 (10.4%) | 1.152 | 0.283 |
| No | 1,542 (90.8%) | 1,085 (91.3%) | 457 (89.6%) |  |  |
| **Influenza vaccine uptake in 20-21 season** |  |  |  |  |  |
| Yes | 573 (33.7%) | 401 (33.7%) | 172 (33.7%) | 0.000 | 1.000 |
| No | 1,126 (66.3%) | 788 (66.3%) | 338 (66.3%) |  |  |
| **Parents** |  |  |  |  |  |
| **Relationship with child** |  |  |  |  |  |
| Mother | 1,306 (76.9%) | 915 (77.0%) | 391 (76.7%) | 0.017 | 0.897 |
| Father | 393 (23.1%) | 274 (23.0%) | 119 (23.3%) |  |  |
| **Age group** |  |  |  |  |  |
| < 26 years | 70 (4.1%) | 52 (4.4%) | 18 (3.5%) | 2.181 | 0.702 |
| 26-30 years | 485 (28.5%) | 343 (28.8%) | 142 (27.8%) |  |  |
| 31-35 years | 746 (43.9%) | 522 (43.9%) | 224 (43.9%) |  |  |
| 36-40 years | 324 (19.1%) | 218 (18.3%) | 106 (20.8%) |  |  |
| ≥41 years | 74 (4.4%) | 54 (4.5%) | 20 (3.9%) |  |  |
| **Educational level** |  |  |  |  |  |
| Junior high school or below | 176 (10.4%) | 118 (9.9%) | 58 (11.4%) | 5.525 | 0.137 |
| High school graduate or equivalent | 334 (19.7%) | 221 (18.6%) | 113 (22.2%) |  |  |
| College or equivalent | 1,077 (63.4%) | 775 (65.2%) | 302 (59.2%) |  |  |
| Master's Diploma or above | 112 (6.6%) | 75 (6.3%) | 37 (7.3%) |  |  |
| **Annual household income** |  |  |  |  |  |
| <50,000 RMB | 104 (6.1%) | 70 (5.9%) | 34 (6.7%) | 2.774 | 0.428 |
| 50,000-99,999 RMB | 459 (27.0%) | 312 (26.2%) | 147 (28.8%) |  |  |
| 100,000-149,999 RMB | 441 (26.0%) | 306 (25.7%) | 135 (26.5%) |  |  |
| ≥150,000 RMB | 695 (40.9%) | 501 (42.1%) | 194 (38.0%) |  |  |
| **Healthcare occupation** |  |  |  |  |  |
| Yes | 108 (6.4%) | 78 (6.6%) | 30 (5.9%) | 0.275 | 0.600 |
| No | 1,591 (93.6%) | 1,111 (93.4%) | 480 (94.1%) |  |  |
| **Vaccine hesitancy** |  |  |  |  |  |
| High | 109 (6.4%) | 78 (6.6%) | 31 (6.1%) | 0.138 | 0.710 |
| Low | 1,590 (93.6%) | 1,111 (93.4%) | 479 (93.9%) |  |  |
| **Willingness to influenza vaccine** |  |  |  |  |  |
| Yes | 1,000 (58.9%) | 705 (59.3%) | 295 (57.8%) | 0.310 | 0.578 |
| No | 699 (41.1%) | 484 (40.7%) | 215 (42.2%) |  |  |
| **Convenience to immunization clinic** |  |  |  |  |  |
| Agreement | 1,071 (63.0%) | 749 (63.0%) | 322 (63.1%) | 0.003 | 0.955 |
| Disagreement | 628 (37.0%) | 440 (37.0%) | 188 (36.9%) |  |  |
| **Satisfaction to immunization clinic service** |  |  |  |  |  |
| Agreement | 1,654 (97.4%) | 1,159 (97.5%) | 495 (97.1%) | 0.242 | 0.623 |
| Disagreement | 45 (2.6%) | 30 (2.5%) | 15 (2.9%) |  |  |


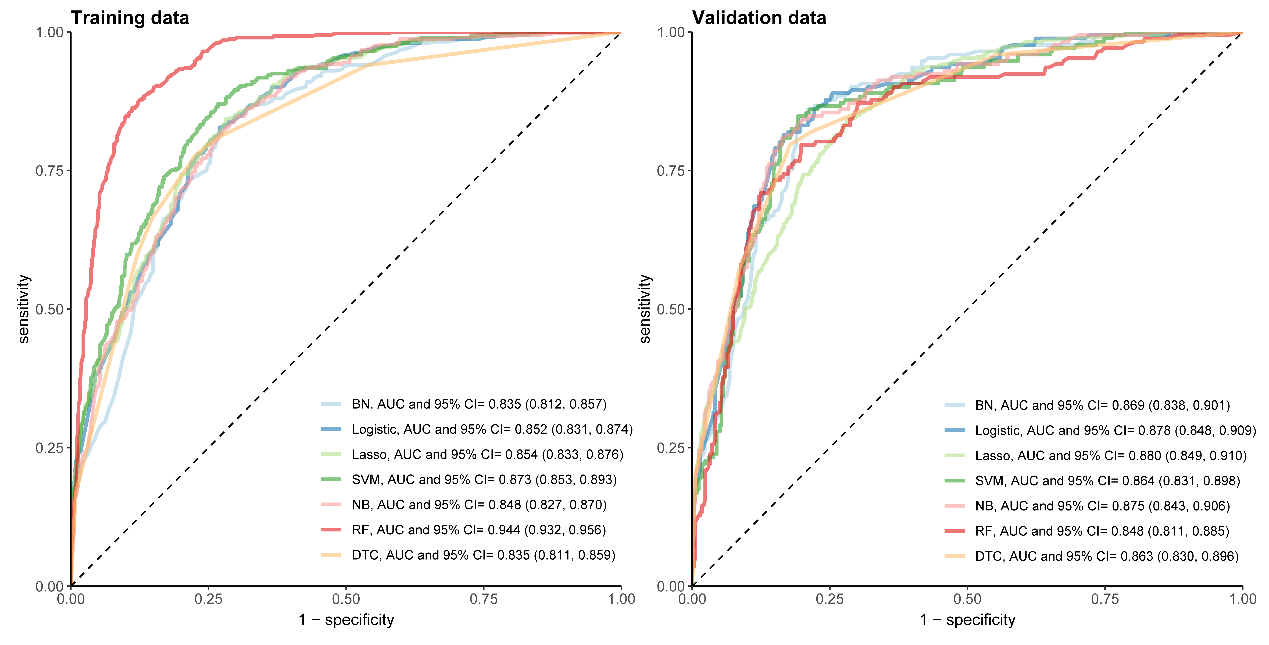


**Figure S4. Receiver operating characteristic curves of various prediction models (children aged less than six months on September, 2019)**

**Table S8. Performance of model (children aged less than six months on September, 2019)**

| **Model** | **Accuracy** | **Precision** | **Recall** | **F1 score** | **Cohen's kappa** |
| --- | --- | --- | --- | --- | --- |
| Training data |  |  |  |  |  |
| Bayesian network | 0.773 | 0.664 | 0.661 | 0.663 | 0.491 |
| Logistic regression | 0.765 | 0.644 | 0.676 | 0.659 | 0.480 |
| Lasso regression | 0.770 | 0.655 | 0.676 | 0.665 | 0.490 |
| Support vector machine | 0.793 | 0.712 | 0.648 | 0.679 | 0.527 |
| Naïve Bayes | 0.771 | 0.645 | 0.716 | 0.678 | 0.502 |
| Random forest | 0.881 | 0.832 | 0.813 | 0.822 | 0.733 |
| Decision tree classifier | 0.780 | 0.720 | 0.571 | 0.637 | 0.483 |
| Validation data |  |  |  |  |  |
| Bayesian network | 0.804 | 0.731 | 0.663 | 0.695 | 0.551 |
| Logistic regression | 0.814 | 0.755 | 0.663 | 0.706 | 0.570 |
| Lasso regression | 0.810 | 0.755 | 0.645 | 0.696 | 0.510 |
| Support vector machine | 0.800 | 0.743 | 0.622 | 0.677 | 0.534 |
| Naïve Bayes | 0.816 | 0.741 | 0.698 | 0.719 | 0.582 |
| Random forest | 0.816 | 0.760 | 0.663 | 0.708 | 0.574 |
| Decision tree classifier | 0.796 | 0.774 | 0.558 | 0.649 | 0.510 |


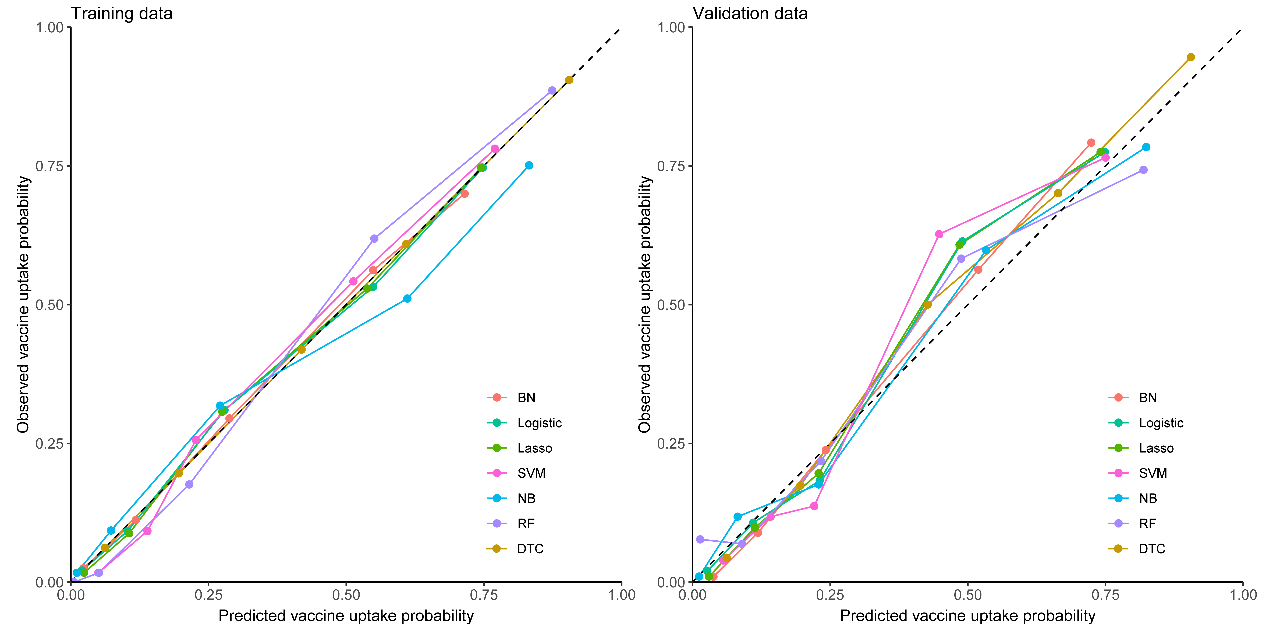


**Figure S5. Calibration Plots for training and validation dataset (children aged less than six months on September, 2019)**

**Table S9. Characteristics of participants (children aged six months to five years)**

| **Characteristic** | **All data**  **(n=1,982)** | **Training data (n=1,388)** | **Validation data (n=594)** | **χ^2^** | **P value** |
| --- | --- | --- | --- | --- | --- |
| **Children** |  |  |  |  |  |
| **Age group** |  |  |  |  |  |
| 6 months-2 years | 1,229 (62.0%) | 849 (61.2%) | 380 (64.0%) | 1.390 | 0.238 |
| 3-5 years | 753 (38.0%) | 539 (38.8%) | 214 (36.0%) |  |  |
| **Sex** |  |  |  |  |  |
| Male | 1,009 (50.9%) | 716 (51.6%) | 293 (49.3%) | 0.849 | 0.357 |
| Female | 973 (49.1%) | 672 (48.4%) | 301 (50.7%) |  |  |
| **Firstborn** |  |  |  |  |  |
| Yes | 1,269 (64.0%) | 866 (62.4%) | 403 (67.8%) | 5.371 | 0.020 |
| No | 713 (36.0%) | 522 (37.6%) | 191 (32.2%) |  |  |
| **Prior influenza vaccine uptake** |  |  |  |  |  |
| Yes | 1,888 (95.3%) | 66 (4.8%) | 28 (4.7%) | 0.002 | 0.968 |
| No | 94 (4.7%) | 1,322 (95.2%) | 566 (95.3%) |  |  |
| **Influenza vaccine uptake in 20-21 season** |  |  |  |  |  |
| Yes | 478 (24.1%) | 335 (24.1%) | 143 (24.1%) | 0.001 | 0.977 |
| No | 1,504 (75.9%) | 1,053 (75.9%) | 451 (75.9%) |  |  |
| **Parents** |  |  |  |  |  |
| **Relationship with child** |  |  |  |  |  |
| Mother | 1,481 (74.7%) | 1,033 (74.4%) | 448 (75.4%) | 0.219 | 0.640 |
| Father | 501 (25.3%) | 355 (25.6%) | 146 (24.6%) |  |  |
| **Age group** |  |  |  |  |  |
| <26 years | 159 (8.0%) | 107 (7.7%) | 52 (8.8%) | 5.226 | 0.265 |
| 26-30 years | 749 (37.8%) | 514 (37%) | 235 (39.6%) |  |  |
| 31-35 years | 761 (38.4%) | 536 (38.6%) | 225 (37.9%) |  |  |
| 36-40 years | 255 (12.9%) | 184 (13.3%) | 71 (12.0%) |  |  |
| ≥41 years | 58 (2.9%) | 47 (3.4%) | 11 (1.9%) |  |  |
| **Educational level** |  |  |  |  |  |
| Junior high school or below | 215 (10.8%) | 146 (10.5%) | 69 (11.6%) | 2.129 | 0.546 |
| High school graduate or equivalent | 385 (19.4%) | 280 (20.2%) | 105 (17.7%) |  |  |
| College or equivalent | 1,245 (62.8%) | 869 (62.6%) | 376 (63.3%) |  |  |
| Master's Diploma or above | 137 (6.9%) | 93 (6.7%) | 44 (7.4%) |  |  |
| **Annual household income** |  |  |  |  |  |
| <50,000 RMB | 132 (6.7%) | 89 (6.4%) | 43 (7.2%) | 1.915 | 0.590 |
| 50,000-99,999 RMB | 574 (29.0%) | 414 (29.8%) | 160 (26.9%) |  |  |
| 100,000-149,999 RMB | 516 (26.0%) | 357 (25.7%) | 159 (26.8%) |  |  |
| ≥150,000 RMB | 760 (38.3%) | 528 (38.0%) | 232 (39.1%) |  |  |
| **Healthcare occupation** |  |  |  |  |  |
| Yes | 130 (6.6%) | 94 (6.8%) | 36 (6.1%) | 0.344 | 0.558 |
| No | 1,852 (93.4%) | 1,294 (93.2%) | 558 (93.9%) |  |  |
| **Vaccine hesitancy** |  |  |  |  |  |
| High | 132 (6.7%) | 97 (7.0%) | 35 (5.9%) | 0.804 | 0.370 |
| Low | 1,850 (93.3%) | 1,291 (93.0%) | 559 (94.1%) |  |  |
| **Willingness to influenza vaccine** |  |  |  |  |  |
| Yes | 1,099 (55.4%) | 763 (55.0%) | 336 (56.6%) | 0.428 | 0.513 |
| No | 883 (44.6%) | 625 (45.0%) | 258 (43.4%) |  |  |
| **Convenience to immunization clinic** |  |  |  |  |  |
| Agreement | 1,249 (63.0%) | 867 (62.5%) | 382 (64.3%) | 0.608 | 0.436 |
| Disagreement | 733 (37.0%) | 521 (37.5%) | 212 (35.7%) |  |  |
| **Satisfaction to immunization clinic service** |  |  |  |  |  |
| Agreement | 1,939 (97.8%) | 1,364 (98.3%) | 575 (96.8%) | 4.233 | 0.040 |
| Disagreement | 43 (2.2%) | 24 (1.7%) | 19 (3.2%) |  |  |


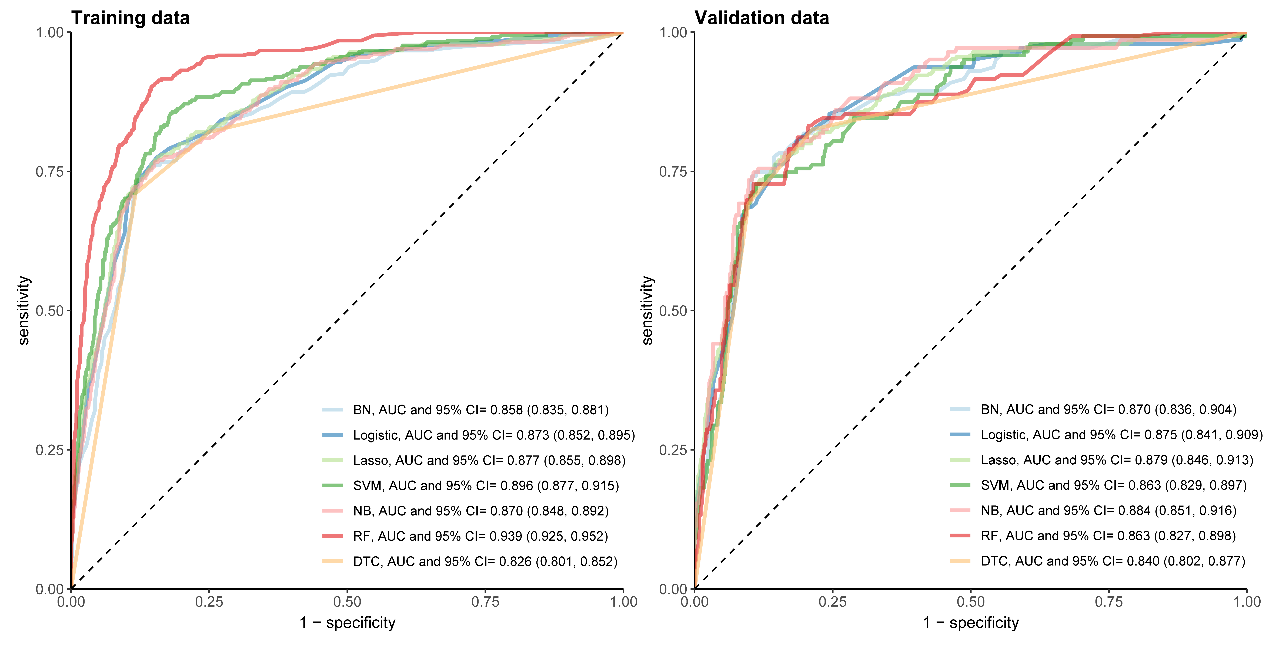


**Figure S6. Receiver operating characteristic curves of various prediction models (children aged six months to five years)**

**Table S10. Performance of model (children aged six months to five years)**

| **Model** | **Accuracy** | **Precision** | **Recall** | **F1 score** | **Cohen's kappa** |
| --- | --- | --- | --- | --- | --- |
| Training data |  |  |  |  |  |
| Bayesian network | 0.843 | 0.656 | 0.734 | 0.693 | 0.588 |
| Logistic regression | 0.845 | 0.667 | 0.716 | 0.691 | 0.588 |
| Lasso regression | 0.847 | 0.672 | 0.710 | 0.691 | 0.589 |
| Support vector machine | 0.849 | 0.678 | 0.710 | 0.694 | 0.593 |
| Naïve Bayes | 0.849 | 0.689 | 0.687 | 0.688 | 0.588 |
| Random forest | 0.885 | 0.778 | 0.731 | 0.754 | 0.679 |
| Decision tree | 0.841 | 0.659 | 0.710 | 0.684 | 0.578 |
| Validation data |  |  |  |  |  |
| Bayesian network | 0.854 | 0.689 | 0.713 | 0.701 | 0.604 |
| Logistic regression | 0.854 | 0.706 | 0.671 | 0.688 | 0.593 |
| Lasso regression | 0.855 | 0.711 | 0.671 | 0.691 | 0.596 |
| Support vector machine | 0.854 | 0.697 | 0.692 | 0.695 | 0.598 |
| Naïve Bayes | 0.860 | 0.727 | 0.671 | 0.698 | 0.607 |
| Random forest | 0.843 | 0.702 | 0.608 | 0.652 | 0.551 |
| Decision tree | 0.852 | 0.692 | 0.692 | 0.692 | 0.595 |


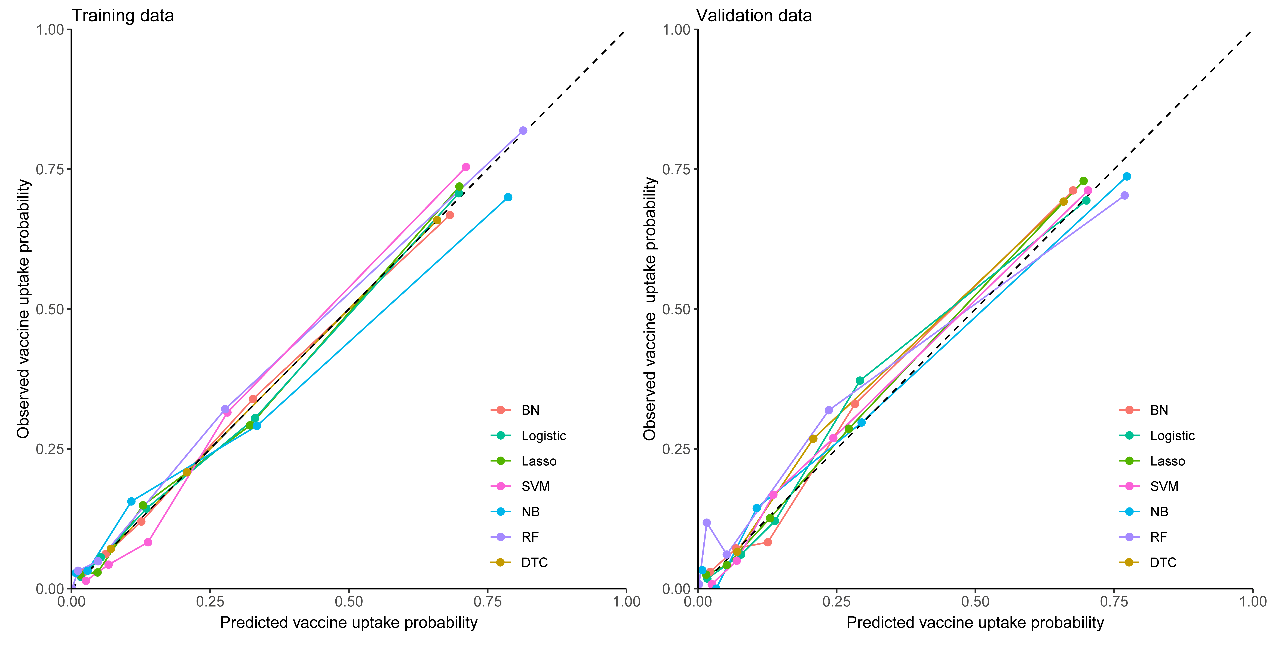


**Figure S7. Calibration Plots for training and validation dataset (children aged six months to five years)**
